# Neurosarcoidosis: The Presentation, Diagnosis and Treatment Review of Two Cases

**DOI:** 10.3390/life14010069

**Published:** 2023-12-31

**Authors:** Maamoun Basheer, Hamd Waked, Helana Jeries, Olga Azrilin, Dan Paz, Nimer Assy, Mohammad E. Naffaa, Samih Badarny

**Affiliations:** 1 Internal Medicine Department, Galilee Medical Center, Nahariya 221001, Israel; nimera@gmc.gov.il; 2 Neurology Department, Galilee Medical Center, Nahariya 221001, Israel; olgaa@gmc.gov.il (O.A.); samihb@gmc.gov.il (S.B.); 3 Rheumatology Unit, Galilee Medical Center, Nahariya 221001, Israel; helanaj@gmc.gov.il; 4 Radiology Department, Galilee Medical Center, Nahariya 221001, Israel; danp@gmc.gov.il (D.P.); mohammadn@gmc.gov.il (M.E.N.); 5 The Azrieli Faculty of Medicine, Bar-Ilan University, Safed 1311502, Israel

**Keywords:** neurosarcoidosis, anti-TNFα agents, infliximab, nervous system, effectiveness, safety

## Abstract

Sarcoidosis is a chronic granulomatous disease of unknown cause characterized by the presence of non-caseating granulomas. The disease can affect any organ including the nervous system. Neurosarcoidosis occurs in about 5% patients with sarcoidosis. The clinical presentation of neurosarcoidosis is varied, and it can involve the brain, spinal cord and peripheral nervous system, separately or in different combinations. The diagnosis of neurosarcoidosis is challenging, as biopsies from the nervous system are not readily available. Anti-TNFα agents are becoming one of the cornerstone treatments for neurosarcoidosis. In this case-based review, we discuss two cases of neurosarcoidosis with different clinical presentations. The first patient presented with confusion, while the second presented with walking difficulty and neurogenic bladder. Both patients were treated with methylprednisolone pulse therapy with rapid, but non-complete, improvement. Therefore, infliximab was initiated in both cases with subsequent improvement in the clinical manifestations and imaging findings, emphasizing the effectiveness and safety of infliximab in cases of severe neurosarcoidosis. In conclusion, the goal of neurosarcoidosis management is to prevent organ system damage and minimize the toxic cumulative adverse effects of glucocorticoid use. In this case-based review we discuss the various presentations, the diagnosis and the treatment of neurosarcoidosis.

## 1. Introduction

Sarcoidosis is a multisystemic inflammatory disease characterized by the formation of non-caseating granulomas in various organ systems, mainly in the lungs and lymphatic system [1,2]. Although the pathogenesis of sarcoidosis is not yet fully understood, environmental and genetic factors may contribute to its pathogenesis and lead to an exaggerated granulomatous response [1,2]. Sarcoidosis occurs worldwide and affects all races and age groups [1]. The clinical manifestation, natural course and prognosis of sarcoidosis vary widely [1,2,3,4]. 

Any tissue can be affected, although the lungs, skin, eyes and liver are the most commonly affected [1,2,3]. The lymphatic system is almost always affected. At the onset of the disease, the lungs are affected in 95% of cases, and multisystem disease is present in about half of cases [1,2,3,4,5]. Neurological symptoms represent the first defining manifestation of sarcoidosis in almost 50% of cases [6,7]. Neurosarcoidosis occurs in 5–10% of patients with sarcoidosis, and these rates are not influenced by race or gender [3,4,5]. The clinical manifestations of neurosarcoidosis are also heterogeneous, as granulomas can affect any part of the brain. Cranial neuropathy is the most common manifestation, with the facial nerves being the most commonly affected [6,7]. 

The purpose of this case-based review is to discuss the various presentations, the diagnosis and the treatment of neurosarcoidosis. 

## 2. Cases Presentation

### 2.1. Case 1

**Figure 1:**
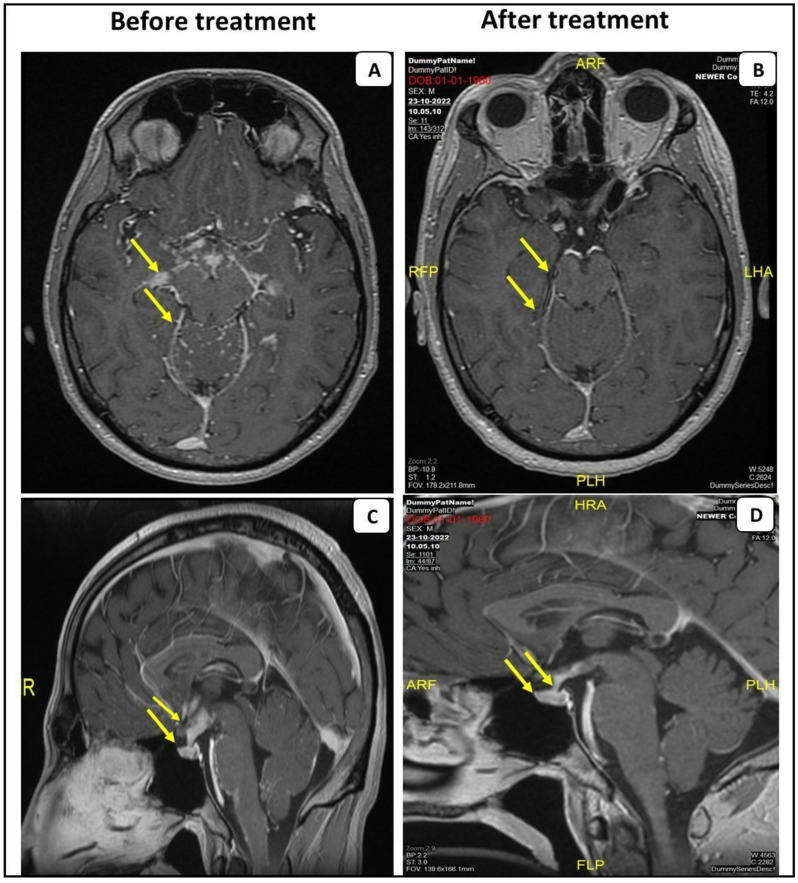
MRI scans with contrast of the first patient before and after the anti-TNF-α treatment. Post-gadolinium T1 MRI from the patient with probable NS, demonstrating leptomeningeal enhancement predominantly affecting the basal cistern of the brain and leptomeningeal spread within the folia of the cerebellum (**A**). Sagittal post-gadolinium T1 MRI demonstrates abnormal contrast enhancement and swelling of the pituitary gland (arrowhead) (**C**). Anti-TNF-α treatment resolved these findings (**B**,**D**).

A 34-year-old healthy male, admitted to the emergency room (ER) due to a confusional state. A month prior to his admission, he complained about headaches, decreased sexual function and weight loss. Brain computed tomography (CT) without contrast demonstrated a hypodense process in the pituitary gland and above it on the right side, producing pressure in the same area. Consequently, brain magnetic resonance imaging (MRI) was performed showing a hypothalamic lesion involving the pituitary stalk, accompanied by secondary leptomeningeal spread mainly to the brain and the basal cisterns (Figure 1A,C). The differential diagnosis of the radiologic findings included neurosarcoidosis, tuberculosis or metastasis. The patient underwent the following workup: An electroencephalogram (EEG) on alertness was slightly disturbed, due to slowing over the frontal area. A Lumbar puncture (LP) showed 98 white blood cells, the majority of which were lymphocytes, a protein level of 330 mg/dL and a glucose level of 30 mg/dL. A BIOFIRE test, a detector panel of a wide range of viruses and bacteria in the cerebrospinal fluid (CSF), was negative. Syphilis serology was negative. Cytology tests showed small lymphocytes and a few histiocytes. Polymerase chain reaction (PCR) for tuberculosis was negative as well. Vitamin B12 and thyroid stimulating hormone (TSH) were normal. Human immunodeficiency virus (HIV) was negative. Synacthen test was also normal. The patient’s visual fields test was normal. 

Chest and abdominal CTs were negative for malignancy. Mediastinal lymphadenopathy was demonstrated. Endobronchial ultrasound (EBUS) examination and sampling of the lymph node was performed. The biopsy showed columnar epithelial cells, lymphocytes and multinucleated cells on the surfaces. No malignant cells were observed. Detached tiny fragments of lymphoid tissue with a focus on non-necrotizing granuloma were evident. 

The patient was treated with high-dose methylprednisilone (1000 mg per day for three sequential days). Clinical improvement was observed. He was discharged with a scheduled prednisone tapering along with weekly oral 20 mg/w of methotrexate. Due to residual disease on the following brain MRI, intravenous infliximab was added to treatment, 5 mg/kg (0, 2 and 6 weeks of a loading dose and every 8 weeks for maintenance), with subsequent clinical and imaging improvement (Figure 1B,D). 

### 2.2. Case 2

A 49-year-old male, diagnosed with sarcoidosis one year prior to his current admission. The diagnosis of sarcoidosis was based on chronic dyspnea and a cough, and a mediastinal lymph node biopsy showing non-caseating granuloma. Methotrexate (MTX) was initiated, but was later stopped due to severe side effects and replaced by azathioprine. In the current admission, the patient presented to the ER with left leg weakness, walking difficulty and recurrent falls and confusion for several days. Brain CT without contrast was normal. A lumbar puncture revealed 213 white blood cells, 988 mg/dL protein and 35 mg/dL glucose. Due to the patient’s immunocompromised status, treatment with acyclovir, ceftriaxone and vancomycin was initiated. The CSF fluid was negative for micro-organisms and CSF cultures were negative. West Nile virus, HIV, VDRL and PCR for herpes and varicella zoster virus (VZV), tuberculosis (TB), non-tubercles mycobacteria, Nocardia and JC virus were all negative. Anti-viral and anti-bacterial therapy were withheld. MRI of the brain was ambiguous and EEG was normal. Due to proximal weakness in the left leg, an electromyogram (EMG) was performed showing left-sided L4-5 radiculopathy. A lumbar spine CT showed a bulging disc at L2-3 on the right (which did not explain the patient’s complaints). 

**Figure 2:**
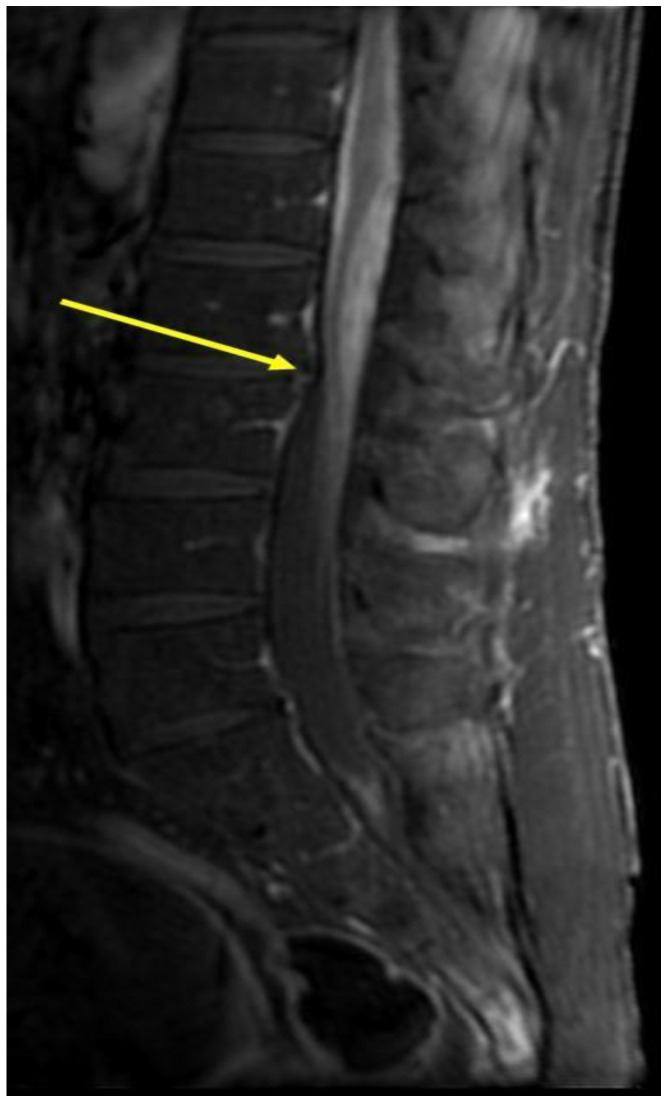
MRI scans with contrast of the second patient before the anti-TNF-α treatment. Spinal MRI of the second patient. Sagittal post-gadolinium T1 MRI of the lumbar spine demonstrating nodular leptomeningeal enhancement about the cauda equina (arrowhead).

Spinal MRI demonstrated a significant enhancement of the lumbar nerve roots accompanied by an enhancement of the intra-dural CSF space surrounding the spinal cord at the level of the conus medullaris (Figure 2). Angiotensin converting enzyme (ACE) levels in the CSF were very high (159,000 U/L), which may confirm the diagnosis of neurosarcoidosis. Methylprednisolone pulse therapy was initiated with subsequent clinical improvement. Later, the patient was transferred to the rehabilitation department with a plan for prednisone tapering down. Because of the previous failure on methotrexate (due to elevated liver enzymes) and azathioprine (lack of efficacy), treatment with infliximab was initiated intravenously with the successful tapering down of prednisone along with clinical improvement. 

## 3. Neurological Involvement in Sarcoidosis

Neurosarcoidosis is characterized by inflammation and abnormal cell deposits in any part of the nervous system: the brain, spinal cord or peripheral nerves. 

### 3.1. Cranial Neuropathy

Facial neuropathy accounts for 70% of isolated cranial neuropathies [8]. It is unilateral at the onset of the disease [8]. Optic neuritis caused by demyelination of the optic nerve is less common. Bilateral involvement is rare, but sequential optic neuropathies occur in 30% of cases [9].

The oculomotor (III), trochlear (IV), abducens (VI), trigeminal (V) and vestibulocochlear (VIII) are less commonly affected. Diagnosis is difficult as imaging is usually normal, but there may be nerve enhancement [10]. Inflammatory masses in the orbit, orbital apex and cavernous sinus can cause diplopia, trigeminal sensory disturbances, pain and proptosis [10].

### 3.2. Peripheral Neuropathy

Peripheral neuropathy accounts for 10–14% of cases [11,12]. The symptoms are sensorimotor or purely sensory. Electrophysiologic studies suggest a predominantly axonal pathology, although conduction slowing, focal conduction block and multifocal conduction block may occur. Mononeuritis multiplex and asymmetric neuropathy may also occur [11,12,13,14], in particular, radial and ulnar neuropathy may occur in some cases.

### 3.3. Pituitary and Hypothalamic Involvement

The involvement of the hypothalamus has been mentioned in the literature since the first reports of neurosarcoidosis. Endocrine symptoms are usually present, especially polydipsia [15,16]. Most patients present with gradual pan-hypopituitarism, while others have endocrine involvement as part of increasing leptomeningitis [15,16]. Endocrine investigations usually reveal gonadotropin and thyrotropin deficiency, diabetes insipidus and corticoadrenal insufficiency [15,16].

### 3.4. Pachymeningitis

Dural inflammation can develop in any part of the cranial cavity, in the basal regions and in the convexity. About 50% of the cases are mass lesions with focal neurological symptoms. The remaining cases have a multifocal, plaque-like appearance that can be very widespread. The involvement of the cavernous sinus and apex orbitals might cause pain, diplopia and optic neuropathy. The CSF is mostly active, but usually not correlated with disease activity [17].

### 3.5. Leptomeningitis

Many cases show features of invasive and destructive meningoencephalitis. Two thirds of cases show signs of dysfunction of the diencephalon and hydrocephalus, while one third show signs of brainstem involvement and associated hydrocephalus. The basal meninges are more frequently affected than the convex meninges. The disease course is subacute but may rapidly progress to severe encephalopathy [18]. Imaging is usually abnormal in leptomeningitis, and the CSF demonstrates a high concentration of proteins and cells and a low CSF/serum glucose ratio [18].

### 3.6. Vascular Involvement

Early pathologic descriptions showed that the predominant pathologic process in neurosarcoidosis is granulomatous inflammation within the leptomeninges that spreads to the underlying parenchyma [19]. The walls of small- and medium-sized arteries show epithelioid cell invasion leading to an inflammatory reaction with the destruction of the inner elastic lamina and fibrosis, resulting in occlusion or the narrowing of the vessel lumen. However, infarction of the adjacent parenchyma has only been noted in one report [19]. Vascular involvement does occur, but the small number of available case reports suggests that it rarely has a direct clinical consequence.

## 4. Diagnosis of Neurosarcoidosis 

The finding of bilateral adenopathy of the hilus with right paratracheal involvement on the chest X-ray makes sarcoidosis very likely. However, in patients with suspected neurosarcoidosis, a careful evaluation of systemic manifestations and neurological examination is required, since the diagnosis of neurosarcoidosis is not based on neurologic features alone [1,2,3,4].

The recommended diagnostic criteria for neurosarcoidosis of the central and peripheral nervous system are categorized into three groups: possible, probable and definite [8,17,18], as shown in Table 1. Possible neurosarcoidosis is determined when clinical findings and MRI, CSF and EMG findings are consistent with granulomatous inflammation of the nervous system and other causes are excluded by detailed investigations, but there is no pathologic confirmation of granulomatous disease [8,17,18]. Probable neurosarcoidosis is diagnosed when clinical findings as well as the MRI, CSF and EMG findings are compatible with granulomatous inflammation of the nervous system (and other causes are excluded by detailed investigations) and there is a non-neurologic pathologic confirmation of granulomatous disease compatible with sarcoidosis. Definite neurosarcoidosis is diagnosed when clinical findings and MRI, CSF and EMG findings are compatible with granulomatous inflammation of the nervous system (and other causes are excluded by detailed investigations) and the nervous system pathology is consistent with neurosarcoidosis [8,18]. 

MRI of the brain is the most important imaging procedure for the assessment of neurosarcoidosis. It shows abnormal findings in one third of patients with cranial neuropathy that is not optic neuropathy and in 100% of patients with central neurologic disease [8,17,18,19,20]. It is important to administer a contrast agent, as contrast enhancement is often the only abnormality and only a small minority show no enhancement. Meningeal enhancement correlates with the location of the disease. In leptomeningeal disease, there is often an enhancement and edema of the underlying cortex and white matter, and some patients show an enhancement of the affected vessel walls. Patients with progressive spinal cord disease may show no abnormalities or atrophy, and patients with vascular involvement, as mentioned above, may only show perfusion abnormalities [8,17,18,19,20].

Fluorodeoxyglucose positron emission tomography/computed tomography (FDG PET/CT) may be useful in cases where other imaging modalities have been unsuccessful in detecting sarcoidosis activity. FDG PET/CT is mainly used to detect extraneural localizations and to identify extraneural biopsy sites [17,18,19,20].

The CSF is almost always active in untreated neurosarcoidosis. The protein level is elevated and lymphocytosis is mostly found. The CSF/plasma glucose ratio is reduced and the CD4/CD8 ratio is elevated. Interleukin 6 (IL-6) and IL-10 concentrations are also elevated in active disease [20]. The ACE level is not a biomarker because it increases in proportion to CSF proteins and is elevated in many inflammatory and infective diseases in which the protein content is elevated [20].

### 4.1. Treatment 

The goal of the treatment of neurosarcoidosis is to reduce or prevent damage to organ systems from the deleterious effects of granulomatous inflammation and to minimize the toxic effects of long-term glucocorticoid treatment [18,21,22,23,24,25,26,27,28,29,30,31,32,33,34,35,36,37,38]. In most patients with CNS sarcoidosis, and in many with large-fiber peripheral nervous system involvement, immunosuppression is indicated early to minimize neurologic damage and disability [21,22,23,24,25,26,27,28,29,30]. Treatments that focus on symptom management and rehabilitation are also important. The first-line therapy is corticosteroids, but approximately 25% of these patients progress from first-line therapy to second- or third-line therapy [21]. Given the safety and toxicity concerns associated with glucocorticoid exposure, early steroid-sparing therapy should be considered [21]. Second-line therapies include methotrexate, azathioprine, hydroxychloroquine, mycophenolate mofetil and cyclosporine A. Anti-TNF-alpha and cyclophosphamide are the third-line therapies. In severe cases, when there is an incomplete response to glucocorticoids, or when there is particular concern about the glucocorticoid toxicity risk, the earlier use of TNF inhibitors is increasingly considered [21,22].

In 2017, a multi-institutional study reported a benefit of the use of infliximab for CNS sarcoidosis [30]. A favorable clinical response was seen in 77% (29% of whom achieved complete remission) and a favorable MRI response in 82% (complete remission in 44%) of patients [29,30]. Recent evidence indicates that other anti TNF agents, such as adalimumab, may also be effective in neurosarcoidosis [30]. This treatment should be continued for a few to several years in patients with a history of severe disease. When discontinuing a TNF inhibitor, it is important to monitor the patient clinically and via MRI for recurrence, which can occur as early as 3–6 months after treatment cessation [30,31,32]. The studies evaluating the effect of a TNF inhibitor on neurosarcoidosis are summarized in Table 2.

Adverse effects of TNF-alpha inhibition include leukopenia, elevated liver enzymes, infusion-related reactions, infections (including reactivation of herpes zoster and latent tuberculosis and new infections with fungi such as histoplasmosis), hypersensitivity reactions, malignancy, inflammatory demyelination of the CNS and rarely progressive multifocal leukoencephalopathy [31,32]. Paradoxical granulomatous reactions, particularly etanercept, have been reported. Immunogenicity, due to neutralizing antibodies, to infliximab occurs in a minority of patients and is associated with infusion reactions and decreased efficacy [31,32].

Prior to the initiation of anti-TNF inhibitors, latent TB should be ruled out using the Mantoux test or the interferon gamma release assay (IGRA) and a chest X-ray. If latent TB is detected, active TB should be ruled out and prophylactic treatment should be administered. Hepatitis B and C and HIV should be ruled out as well and the patients should be vaccinated according to guidelines, including pneumococcal, influenza and herpes zoster virus vaccines [25]. 

All patients should be monitored for their response to the treatment. For many patients, the goal may be complete remission of the neuroinflammatory response. In others, suppression of the inflammatory response, even if complete remission is not achieved, may be the right balance between efficacy and the safety of the treatment used. Monitoring includes medical history, physical examination and usually MRI with and without contrast, which can detect a worsening or recurrence of the disease even before clinical symptoms appear [21,22]. If the MRI shows no abnormal findings or if the clinical symptoms and MRI findings are not consistent (especially if the CSF examination has shown intrathecal inflammation), a repeat CSF examination may be clinically helpful to monitor disease activity and confirm remission [21,22].

### 4.2. Prognosis 

The treatment response is variable [23,24,25,26,27]. A systematic review showed that the mortality seen in the majority of the studies ranges from 0 to 33% [23]. Total remission was achieved in 27% of patients, incomplete remission in 32%, stable disease in 24% and deterioration in 6% of these patients. 

## 5. Summary

Neurosarcoidosis has a heterogeneous clinical presentation. About 5% of patients with sarcoidosis develop neurosarcoidosis. Some of these patients present with confusion and stroke manifestations. Other clinical manifestations such as cranial neuropathy, spinal cord inflammation, peripheral neuropathy or myopathy or (chronic) meningitis may be part of the presentation [1,2,3,4,5]. The main goal of the treatment of neurosarcoidosis is to reduce or prevent damage to organ systems due to the deleterious effects of granulomatous inflammation [20,21,22,23,24,25,26,27]. In certain circumstances, such as mild or transient disease, immunosuppression may not be necessary, but in most patients with CNS sarcoidosis and in many with large-fiber involvement of the peripheral nervous system, immunosuppression is indicated early to minimize neurologic damage and disability. Although new treatments such as TNF-alpha antagonists are increasingly used, the mortality rate in patients with neurosarcoidosis remains at 5% and about one third of patients do not experience significant clinical improvement with treatment [21,22,28,29].

In summary, this case-based review shows that the diagnosis of neurosarcoidosis can be challenging, especially when it presents as a stroke. Anti-TNF-α agents have become a cornerstone of the treatment of moderate to severe cases. 

## Figures and Tables

**Table 1:** The criteria for possible, probable and definitive neurosarcoidosis.

Nervous system pathology is consistent with neurosarcoidosis	Non-nervous system pathology is consistent with sarcoidosis	Clinical, MRI, CSF and EMG findings are compatible with granulomatous inflammation of the nervous system	
	There is no pathologic confirmation of granulomatous disease	Yes (MRI, CSF and/or EMG/NCS findings are typical of granulomatous inflammation)	Possible
	Yes (pathologic confirmation of systemic granulomatous disease consistent with sarcoidosis)	Yes (MRI, CSF and/or EMG/NCS findings are typical of granulomatous inflammation)	Probable
Yes	Yes or No (pathologic confirmation of systemic granulomatous disease consistent with sarcoidosis)	Yes (MRI, CSF and/or EMG/NCS findings are typical of granulomatous inflammation)	Definite

**Table 2:** Main features of patients with neurosarcoidosis treated with anti-TNF-α drugs.

Reference	Age/Sex	Neuroanatomic Location	Extra-Neurological Effect	Biopsy Location	Anti-TNF-α Therapeutic Effect
M W O’Reilly et al., 2013 [31]	22/Male	Hypothalamic infiltration	Adipsic diabetes insipidus	Excisional cervical lymph node biopsy	Infliximab: radiological disease remission and complete recovery of osmoregulated thirst appreciation
Lakshman Arcot Jayagopal et al., 2023 [32]	56/Male	Multi-focal enhancing and longitudinally extensive lesion with dorsal subpial enhancement	Dysesthesia in bilateral arms and legs up to the buttocks and gait difficulties.	Biopsy of a pulmonary mass showed a granuloma	Infliximab: remission of neurosarcoidosis after immunosuppression
Fleur Cohen Aubart et al., 2017. [33]	1 males (29–53) 7 females (34–51)	All had neurological involvement consisting of meningeal (n = 16), cerebral (n = 10), spinal cord (n = 6) and/or optic nerve (n = 5) involvement		All patients had histologically proven non-caseating granulomas.	At 6 months after the initiation of infliximab, six patients obtained complete remission (33%), ten attained partial remission (56%), and two had stable disease (11%)
Quentin Riller et al., 2019 [34]	11 males (33–51) 9 females (37–52)	Meningeal (n = 15), cerebral (n = 10), spinal cord (n = 9) and/or cranial nerves (n = 5); epilepsy (n = 3); and/or intracranial hypertension (n = 3)		Histological documentation of noncaseating granuloma	Infliximab biosimilar: six patients relapsed during biosimilar treatment
Lorentzen, Anastasia Orlova et al., 2014 [35]		Spinal cord sarcoidosis		Biopsy-proven sarcoidosis	Immediate and dramatic response to infliximab
Daan Fritz et al., 2020 [36]	16 males (33–49), 12 females (33–49)	Cerebral parenchymal localization in 16 (59%), pituitary gland/hypothalamic sarcoidosis in 15 (54%), peripheral nerve involvement in 12 (43%) and chronic meningitis in 11 patients (41%).		Biopsy-proven sarcoidosis	Infliximab led to complete remission in 6 (21%) and improvement in 14 (50%), whereas 7 patients had stable disease (25%), and 1 (4%) deteriorated and died
D. Sofia Villacis-Nunez et al., 2021 [37]	17/Female	Diffuse granulomatous involvement of the pituitary gland	Sarcoidosis of the lacrimal gland	Histology of the right lacrimal gland	Corticosteroids, methotrexate, and adalimumab, with complete radiologic resolution
Samy Metyas et al., 2014 [38]	54/Female	Marrow edema and infiltrates and active demyelination	Lower back pain and bilateral lower extremity weakness with 6-month duration	Lung biopsy was performed	Adalimumab and methotrexate reduced headache completely and showed improvement in spinal mobility and range of motion
Spencer K Hutto et al., 2021 [32]	4 males (42–59), 3 females (42–59)	Spinal cord (4), cerebral parenchyma (1), pituitary (1) and leptomeninges (1). The cauda equina was involved in 2 cases	Erythema nodosum, arthritis and uveitis	Pathologically proven sarcoidosis from mediastinal or hilar lymph node biopsy	50% improved, remission was maintained in 30%, and 20% with active disease remained stable without further worsening

## Data Availability

The data presented in this study are available on request from the corresponding author. The data are not publicly available due to privacy of the patient.
